# Updates on genetics in systemic sclerosis

**DOI:** 10.1186/s41232-021-00167-6

**Published:** 2021-06-15

**Authors:** Yuko Ota, Masataka Kuwana

**Affiliations:** Department of Allergy and Rheumatology, Nippon Medical School Graduate School of Medicine, 1-1-5 Sendagi, Bunkyo-ku, Tokyo, 113-8603-8582 Japan

## Abstract

Systemic sclerosis (SSc) is a complex disease, in which an interaction of genetic and environmental factors plays an important role in its development and pathogenesis. A number of genetic studies, including candidate gene analysis and genome-wide association study, have found that the associated genetic variants are mainly localized in noncoding regions in the expression quantitative trait locus and influence corresponding gene expression. The gene variants identified as a risk for SSc susceptibility include those associated with innate immunity, adaptive immune response, and cell death, while there are only few SSc-associated genes involved in the fibrotic process or vascular homeostasis. Human leukocyte antigen class II genes are associated with SSc-related autoantibodies rather than SSc itself. Since the pathways between the associated genotype and phenotype are still poorly understood, further investigations using multi-omics technologies are necessary to characterize the complex molecular architecture of SSc, identify biomarkers useful to predict future outcomes and treatment responses, and discover effective drug targets.

## Introduction

Systemic sclerosis (SSc) is a complex autoimmune disease with heterogeneous clinical manifestations. The pathogenesis of SSc includes microvasculopathy, chronic inflammation and autoimmunity, and excessive fibrosis in the skin and internal organs, such as the lungs, heart, and gastrointestinal tract [1]. One of the autoimmune features is production of autoantibodies to various nuclear proteins, including centromere/kinetochore, topoisomerase I (topo I), and RNA polymerase III [2]. The primary event in the pathogenesis of SSc is thought to be endothelial injury, followed by aberrant vascular and immune dysregulation, leading to excessive tissue fibrosis [3]. The etiology of SSc is largely unknown, but accumulating evidence has shown that the combination of environmental and genetic factors contributes to the development and heterogeneous expression of the disease. Several environmental factors have been shown to correlate with increased SSc susceptibility, such as the exposure to certain chemical compounds, e.g., silica, organic solvents, dry cleaning detergents, vinyl chloride, and epoxy resin [4–6], and microorganisms such as cytomegalovirus [7]. Nevertheless, during the last decade, a number of genetic markers have been reported to be associated with SSc susceptibility and/or certain SSc subsets. However, the pathways between the associated genotype and phenotype as well as the interplay between the genetic risk and environmental triggers are still poorly understood. This review features updated knowledge of roles of genetic factors in susceptibility and disease expression of SSc.

## Family association studies

Roles of the genetic background in susceptibility of SSc were first examined in familial association studies. Analysis of combined American cohorts involving 703 families found that SSc occurred significantly more frequently in families with SSc (1.6%) than in the general population (0.026%) [8]. In a follow-up study, affected first-degree relatives within multicase SSc families were concordant for SSc-related autoantibodies and human leukocyte antigen (HLA) class II haplotypes than expected by chance [9]. The heritability of the disease is often assessed by the disease concordance in monozygotic twins, and this strategy successfully demonstrated contribution of genetic backgrounds to susceptibility of systemic lupus erythematosus (SLE) [10] and rheumatoid arthritis (RA) [11]. The largest SSc twin study included 42 twin pairs, including 24 monozygotic and 18 dizygotic twins, and found that overall concordance of SSc was as low as 4.7%, and was similar in monozygotic and dizygotic twins [10]. This concordance rate was much lower than those in other autoimmune diseases, ~ 25% in SLE or RA. Consistency for the presence of anti-nuclear antibodies (ANAs) was significantly higher in monozygotic twins compared to dizygotic twins (90% versus 40%), suggesting that genetic background contributes mainly to autoantibody responses, but the underlying genetic backgrounds themselves are not sufficient for development of the disease [12]. In addition, families of SSc patients have an increased risk to develop other autoimmune diseases, such as autoimmune thyroid diseases and SLE, or some of SSc manifestations including Raynaud’s phenomenon and interstitial lung disease (ILD), implicating shared genetic components between SSc and other autoimmune diseases [13].

## Genetic association studies

The types of genetic variation include single-nucleotide polymorphism (SNP), variable number of tandem repeat (VNTR) or microsatellite, and copy-number variation (CNV). Of these, the most frequent genetic variation in human is the SNP, which potentially influences the protein function due to alteration in the amino acid sequence or modifies the gene expression. Two basic approaches used for genetic association studies include the candidate gene approach (CGA) and the genome-wide association study (GWAS). These approaches identify genetic variations and determine the likelihood that the variant occurs more or less frequently in the cases than in the controls. The associations are first tested in a discovery cohort and then are verified in a non-overlapping group of cases and controls for replication. The CGA is hypothesis-driven and is able to analyze all types of genetic variations, primarily based on associations with the potential pathogenic process and associations previously reported in other autoimmune diseases, although it is principally impossible to identify the gene loci that have not been assumed to be associated with the disease. Several genes of interest identified by the CGA in SSc patients are listed in Table 1 [14–41, 43]. The majority of genetic variations identified were SNPs, but there were some microsatellite or CNV associations. The genes analyzed were mainly those involved in innate and acquired immune responses or fibrosis.

**Table 1:** SSc susceptibility genes identified by the CGA

Gene	Genetic polymorphism	Molecular function	References
*PTPN22*	SNP	T cell receptor (TCR) signaling	[14–16]
*BANK1*	SNP	B cell signaling	[17, 18]
*CTGF*	SNP	Fibroblast proliferation and production of extracellular matrix	[19]
*FAM167A-BLK*	SNP	B cell receptor signaling and B cell development	[20]
*IRF5*	SNP	TLR-dependent type I interferon production	[21, 22]
*TNFAIP3*	SNP	Negative feedback regulation of the NF-*κ*B pathway	[26, 27]
*STAT4*	SNP	Induction of T helper 1 cells	[28]
*FAS*	SNP	Apoptosis of a wide variety of cell types	[31]
*TBX21*	SNP	T helper 1 cell differentiation	[32]
*TNFSF4*	SNP	Immune regulation	[15, 33]
*TNIP1*	SNP	Regulation of the NF-κB signaling pathway	[34]
*IRAK1*	SNP	Innate immune signaling	[35]
*KCNA5*	SNP	Potassium voltage-gated channel	[36]
*TNFa13*	VNTR	Modulator of inflammation	[37]
*COL1A2*	VNTR	Component of type I collagen	[38]
*NOS2*	VNTR	Production of a reactive free radical	[39]
*CD19*	VNTR	Regulation of B cell function	[40]
*IRAK1*	SNP	Mediator of innate immune response	[41, 42]
*IL-21*	SNP	Mediator of acquired immune response	[43]
*IL-2RA*	SNP	T cell activation	[43]

Over the past 10 years, GWAS that scans the entire genome for SNPs provides powerful approach to analyze the genetic components of the polygenic diseases in hypothesis-free setting [44]. This strategy enables us to allow identification of new disease-related gene loci and pathways in an unbiased manner. However, GWAS approach often miss unusual or rare variants since most GWAS covers up to 80% of common polymorphisms in the human genome. In addition, SNPs assessed by the GWAS are selected as landmarks of the surrounding SNPs in strong linkage disequilibrium. Therefore, additional analysis including whole genome sequencing, functional assays, and expression analysis in the affected and un-affected tissue is always required to identify the “true” SNPs responsible for disease pathogenesis. In early GWAS conducted in SSc patients, the strongest association identified was found in the HLA class II region on chromosome 6 [45], but it was difficult to identify the responsible gene loci due to the considerable variability in allele distribution among ethnic groups and the complex genetic structure of the HLA system. The GWAS approaches followed by replication studies and functional assay have led to identification of several non-HLA loci as an SSc susceptibility genes (Table 2) [42, 46–66]. It is of note that CGA is less reliable under the viewpoint of statistical significance, while identification of the primary associated variant in the locus is often difficult due to linkage disequilibrium. The majority of robustly replicated SSc susceptibility loci are involved in innate or adaptive immune system, and some were associated with cell death pathways. Additional immune-related genes responsible for SSc susceptibility were identified using the ImmunoChip array, another genotyping platform for SNP genotyping with high-density mapping of 196,524 variants across 186 known risk loci for autoimmune and inflammatory diseases in European Caucasian population [44]. These high-throughput genotyping studies found that most of the SSc-associated immune-related genes were shared among other autoimmune diseases, such as SLE and RA [67]. Interestingly, genes directly involved in the fibrotic process and/or vascular homeostasis were scarcely detected in the GWAS, although subsequent integration of multi-ethnic data and meta-analysis with increased sample size have revealed some candidate genes associated with fibrosis as the SSc-associated genes [52]. The hypothesis-free results from the GWAS support the hypothesis that the genetic background shared by many autoimmune diseases primarily contributes to dysregulated autoimmune responses in patients with SSc but suggest additional indispensable roles of environmental factors and epigenetic influences in the development of SSc.

**Table 2:** Non-HLA SSc susceptibility genes identified by the GWAS

Gene	SNP	Study type	References
Innate immunity			
*IRF4*	rs9328192	GWAS	[42]
*IRF5*	rs4728142, rs10488631, rs10488631, rs3757385, rs109542313, rs2004640, rs12537284, rs2280714	GWAS	[42, 47–50, 46, 51]
*IRF5-TNPO3*	rs36073657, rs12155080	meta-GWAS	[52]
*IRF7*	rs1131665, rs4963128, rs702966	CGA, meta-GWAS	[52, 53]
*IRF8*	rs11642873, rs2280381, rs11117432, rs11644034, rs12711490, rs7202472, rs11117420	GWAS, meta-GWAS,	[47, 50, 53–56, 52, 57]
*TNFAIP3*	rs5029929, rs2230926, rs6932056	GWAS	[47, 50, 56, 58]
*TNIP1*	rs4958881, rs2233287, rs3792783	GWAS, meta-GWAS,	[47, 49, 52, 59]
*TAP2*	rs12538892, rs17500468	ImmunoChip	[51]
*NFKB1*	rs230534	meta-GWAS	[52]
Adaptive immune response			
*TNFSF4*	rs4916334, rs10798269, rs12039904	GWAS	[47, 50, 60]
*TNFSF4-LOC100506023-PRDX6*	rs2022449, rs1857066	meta-GWAS	[52]
*CD247*	rs2056626	GWAS, meta-GWAS	[48, 52, 54, 61]
*CSK*	rs1378942	GWAS, GWAS follow-up	[47, 52, 60]
*PTPN22*	rs2476601	GWAS	[42]
*STAT4*	rs7574865, rs3821236, rs4853458, rs10168266, rs3821236	GWAS, ImmunoChip, meta-GWAS	[46, 49, 48, 50, 52, 54, 55]
*BLK*	rs13277113, rs2736340	GWAS	[47]
IL-12 Signaling Pathway and cytokines			
*IL-12A*	rs7758790, rs589446	GWAS, ImmunoChip	[51, 52]
*TYK2*	rs2304256, rs34536443, rs12720356, rs35018800	ImmunoChip follow-up	[62]
*IL-12RB1*	rs436857, rs2305743, rs8109496, rs11668601	meta-GWAS	[52, 63]
*IL-12RB2*	rs3790566, rs924080, rs3790567	GWAS, meta-GWAS	[52, 64]
Apoptosis, Autophagy Pathway			
*DNASEIL3*	rs35677470	ImmunoChip	[51]
*FLNB-DNASE1L3-PXK*	rs7355798, rs4076852	meta-GWAS	[52]
*ATG5*	rs9373839, rs633724	GWAS, ImmunoChip, meta-GWAS	[42, 47, 51, 52]
*PRDM1*	rs4134466	GWAS	[55]
*GSDMA*	rs3894194	GWAS	[65]
*GSDMB*	rs883770	meta-GWAS	[52]
*NOTCH4*	rs443198	GWAS	[53]
Vascular homeostasis and fibrosis			
*PPARG*	rs310746	GWAS follow-up	[66]
*Other*			
*NAB1*	rs16832798	meta-GWAS	[52]
*DDX6*	rs11217020	meta-GWAS	[52]
*DGKQ*	rs11724804	meta-GWAS	[52]
*POGLUT1-TIMMDC1-CD80-ARHGAP31*	rs9884090	meta-GWAS	[52]
*RAB2A-CHD7*	rs6598008	meta-GWAS	[52]
*TSPAN32, CD81-AS1*	rs2651804	meta-GWAS	[52]
*NUP85-GRB2*	rs1005714	meta-GWAS	[52]

Most of the genetic risk factors for SSc are located in intronic regions, rather than coding regions, and act as regulatory variants modulating the expression of nearby genes, i.e., transcription factor binding sites in expression quantitative trait locus (eQTL) [68]. A recent eQTL analysis in combination with GWAS data on SSc-associated genes successfully identified differentially regulated genes in the affected tissues in SSc patients and candidate genes potentially targeted by approved medications for immune-mediated diseases [69].

## SSc susceptibility genes outside the HLA region

### Genes involved in innate immunity

Type 1 interferon (IFN) is an important mediator of innate immunity often triggered by microbial infection. Over the past several years, there has been increasing evidence of dysregulation of the type 1 IFN pathway in autoimmune diseases, including SLE, dermatomyositis, and SSc [70–72]. Increased expression and activation of type I IFN-inducible genes termed “type I IFN signature” has been observed in peripheral blood and affected skin of SSc patients [70–72]. The GWAS identified transcription factors involved in regulation of type I IFN signaling, such as interferon regulatory factor (*IRF*) 4 [42], *IRF5* [42, 46–51], *IRF7* [52, 53], and *IRF8* [47, 50, 52–57]. Interestingly, these genes were associated with susceptibility of SLE and other autoimmune diseases [73–77]. Since all single markers within the *IRF5* loci failed to detect association signals, disease susceptibility could be regulated by the haplotype within the *IRF5* locus [78]. *IRF5* mediates induction of proinflammatory cytokines such as interleukin (IL)-6, IL-12, IL-23 and tumor-necrosis factor (TNF)-α and defines the phenotype of macrophages. In fact, macrophages carrying the *IRF5* risk allele haplotype have an increased expression of IRF5 protein and pattern recognition receptor-induced Akt2 activation, leading to proinflammatory cytokine production and M1 macrophage polarization [78]. The association between the *IRF5* genotype and SSc patients, especially those with anti-topo I-positive diffuse cutaneous SSc (dcSSc) with ILD, was first reported in French population by CGA [21] and was later replicated in independent studies [46]. One of the *IRF5* SNPs was shown to be useful in predicting a longer survival and preserved lung function [23]. A non-synonymous SNP located in the *IRF7* was associated with SSc with anticentromere antibody (ACA) in the USA and European cohorts [53] and was replicated in a meta-GWAS [52].

Another gene identified by the GWAS in SSc patients includes TNF-α-induced protein 3 (*TNFAIP3*), also known as A20 protein, which negatively regulates the TNF-induced nuclear factor (NF)-κB signaling pathway [26]. Three intronic risk variants that were linked to a decreased expression of A20 protein and one exonic variant were associated with SSc. The risk non-synonymous variant with an amino acid substitution was associated with reduction of activity of A20 protein. In this regard, the decrease of A20 expression by siRNA in foreskin fibroblasts resulted in an enhanced stimulation of collagen and α-smooth muscle actin (α-SMA) gene expression after transforming growth factor-β (TGF-β) stimulation [79], suggesting that impaired A20 activity contributes to increased collagen production mediated by TGF-β. TNFAIP3-interacting protein 1 (*TNIP1*), which regulates TNFAIP3 activity, was also identified as the SSc-associated gene by GWAS in European population [49] and was replicated in a meta-GWAS [52].

### Genes involved in adaptive immune response

TNF ligand superfamily member 4 (*TNFSF4*) encoding the T cell co-stimulatory molecule OX40 ligand was identified as the SSc-associated gene by the GWAS [47, 50, 60], and was replicated in CGA of a large European cohort. On the other hand, CD247 or zeta chain of the T cell receptor (TCR)/CD3 complex was identified as a susceptibility gene for SSc by the GWAS in European population [48], but this association was not replicated by CGA in Chinese cohort [80] and by a trans-ethnic meta-GWAS analysis [55]. Protein tyrosine phosphatase, non-receptor type 22 (*PTPN22*) was identified as the susceptibility gene for SSc by the GWAS [42] as well as for a wide range of autoimmune diseases [81]. This gene encodes the lymphoid tyrosine phosphatase (LYP), which directly interacts with c-Src kinase (CSK) and negatively regulates the TCR/CD3 complex signaling. The association of missense *PTPN22* allele detected in SSc patients disrupts the interaction of LYP with CSK and leads to an increased LYP activity [14–16]. *CSK* was also identified as the susceptibility gene associated with SSc by the GWAS [47, 52, 60]. Interestingly, CSK is known to function not only as a regulator of T cell activation, but also as a regulator of myofibroblast differentiation by modulating the function of Src kinase [82].

Signal transducer activator transcriptional factor 4 (*STAT4*) is one of susceptible genes for many autoimmune diseases [81] and is a transcription factor activated by a variety of cytokines, including type 1 IFN, IL-2, IL-12, IL-23, IL-27, and IL-35. STAT4 was first discovered to be crucial for promoting cellular-mediated immune responses via the differentiation of T helper 1 (Th1) cells through IFN-γ production [83], but is involved in a variety of inflammatory and immune processes. There were some contradictory findings in terms of the *STAT4* SNP associations with SSc susceptibility: association with limited cutaneous SSc (lcSSc), but not with dcSSc in European Caucasian cohorts [28, 46, 48, 49], while association with dcSSc and anti-topo I antibody in Chinese cohort [29]. Nevertheless, the recent GWAS meta-analysis and meta-GWAS confirmed the association of the responsible allele with overall SSc [46, 52, 55]. In addition, a large European study showed the additive effect of the *STAT4* and *IRF5* polymorphisms on susceptibility to SSc and SSc-related ILD [30]. The SSc-susceptible SNPs within the intron of *STAT4* locus are known to be eQTL. The STAT4 protein expression might control susceptibility of tissue fibrosis, since STAT4 knock-out mice were protective against bleomycin-induced dermal fibrosis [84]. The genes involved in B cell differentiation were also identified as SSc susceptible genes. B cell-specific scaffold protein with ankyrin 1 (*BANK1*) [15, 16] encodes a signaling molecule involved in B cell mobilization, and *BLK* encodes a tyrosine kinase crucial for B cell development and signaling. The association of *BANK1* SNPs in SSc susceptibility was revealed by two independent CGA studies [17, 18], and later confirmed by whole-exon sequencing [85]. The association of B lymphocyte kinase (*BLK*) was established in both European and Japanese populations [20, 22] and was confirmed by meta-analysis [86]. The trans-ethnic meta-analysis of GWAS identified B lymphocyte-induced maturation protein 1 (*PRDM1*) [55] as the SSc susceptibility gene.

Three genes in the IL-12 signaling pathway, including the intergenic region of *IL12A* [51, 52], IL-12 receptor B (*IL-12RB*)-1 [52, 63], and *IL-12RB2* [52, 64], were reported to be associated with SSc by GWAS, implying an important role of IL-12-mediated Th1 response in SSc pathogenesis. TYK2 encoding a tyrosine kinase member of the Janus kinase-STAT family, and mediates signaling of IL-12 family cytokines, such us IL-12 and IL-23, and is a common genetic risk factor for several autoimmune diseases, such as RA and SLE [87]. Meta-analysis of ImmunoChip analysis revealed that a common TYK2 missense variant was associated with SSc susceptibility [62]. On the other hand, SNPs within the *IL-21* gene, which plays a critical role in follicular helper T cell differentiation and germinal center formation, was shown to be associated with SSc in European/US Caucasian population by the CGA [43].

### Genes involved in cell death

Deoxyribonuclease 1-like 3 (*DNASE1L3*) plays an important role in DNA fragmentation during apoptosis. The ImmunoChip analysis revealed that the non-synonymous *DNASE1L3* SNP, resulting in diminished DNase activity, was associated with SSc [51]. The GWAS follow-up study identified growth factor receptor-bound protein 10 (*GRB10*) as SSc susceptible gene [54]. GRB10 is an adaptor protein known to interact with a number of tyrosine kinase receptors and signaling molecules and has a potential role in apoptosis regulation. On the other hand, genes associated with autophagy were also detected by GWAS and ImmunoChip study. These included autophagy-related 5 (*ATG5*), which plays a role in assisting in autophagosome elongation and regulating lymphocyte maturation via autophagy [51], and Ras-related protein Rab-2A (*RAB2A*), which is involved in autophagosome clearance [52]. These variants could impair proper functioning of autophagy, leading to endothelial cell stress pathways activation. A meta-GWAS analysis identified the association of SSc susceptibility with *GSDMA/B* encoding gasdermin A/B, which have a potential role in pyroptosis, a highly inflammatory cellular death [52, 55]. The gene encoding neurogenic locus notch homolog protein 4 (NOTCH4), which is involved in cell proliferation, differentiation, and apoptosis, was identified as a SSc susceptibility gene by the GWAS [53]. A recent CGA of large Caucasian and Chinese cohorts found associations of multiple NOTCH4 exonic variants with SSc and/or SSc subtypes [88].

### Genes involved in vascular homeostasis and fibrosis

Only few SSc-associated genes involved in the vascular homeostasis and fibrotic process have been identified the SSc-susceptible gene. The GWAS follow-up analysis identified a SNP located upstream of the gene for the peroxisome proliferator-activated receptor gamma (PPARG) as one of SSc susceptible genes [66]. PPARG was initially identified in adipose tissue and was shown to be an anti-fibrotic effector through suppression of collagen synthesis, myofibroblast differentiation, and other TGF-β-induced fibrotic responses [89]. The genes whose molecular and cellular function has not been investigated in detail in mammalians, such as DDX6, NAB1, and DGKQ, have been identified by meta-GWAS study [52], but additional studies are necessary to clarify roles of these genes in the pathogenic process of SSc. *CAV1* was shown to be associated with SSc susceptibility by the CGA [90]. This gene encodes caveolin 1, which is an inhibitor of tissue fibrosis by suppressing TGF-β signaling. However, this association has not been replicated in independent studies. Dense microsatellite analysis of the HLA region in Japanese SSc patients identified a relationship between a rare variant of retinoid X receptor-beta (*RXRB*) and SSc patients with anti-topo I antibody on the risk haplotype harboring HLA-DPB1*13:01 [91]. *RXRB* plays roles in anti-fibrotic activity through formation of a heterodimer with peroxisome proliferator-activated receptors and 9-cis retinoic acid ligands [92].

## Roles of HLA gene polymorphisms in SSc susceptibility

Since immune dysregulation is one of characteristic pathogenic features of SSc [1], HLA has been examined extensively as one of potential genetic factors. Despite this, HLA associations with susceptibility to SSc were generally weak and inconsistent among studies, owing to diverse distribution of gene polymorphisms among ethnic groups [93]. Twelve different gene loci, including *HLA-B*, *C*, *DRA*, *DRB1*, *DRB5*, *DQA1*, *DQB1*, *DMB*, *DOA*, *DPA1*, *DPB1*, and *DPB2* were reported to be associated with SSc. Of these, *DRB1*, *DQA1*, *DQB1* and *DPB1* loci were extensively analyzed, but it was difficult to identify the responsible gene loci due to strong linkage disequilibrium. One of the most extensive studies enrolling 1300 SSc patients and 1000 controls with Caucasian, African, and Hispanic American backgrounds found that the associated HLA class II alleles were different among ethnic groups, and all associations were not robust [94]. The associations between susceptibility of SSc and the third hypervariable region (HVR) sequences of the *DRB1* gene were also investigated but were again borderline [95]. On the other hand, the HLA region (6p21.3), especially the *HLA-DPB1* and *DPB2*, was consistently identified as the gene region most strongly associated with SSc by GWAS, and this association was most prominent in SSc patients with anti-topo I antibody [45], but it remains controversial if the primarily associated genes were located within HLA or non-HLA genes.

**Fig. 1:**
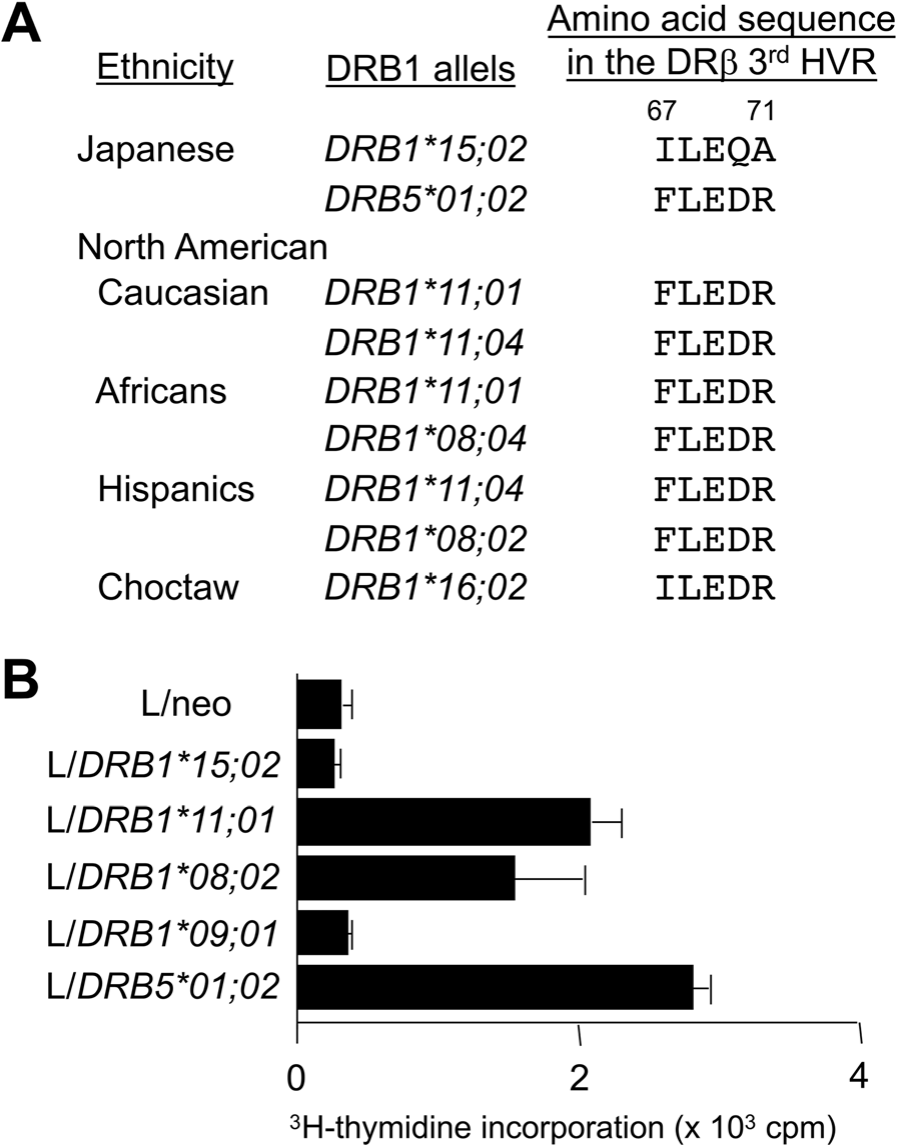
**A** *DRB1* or *DRB5* alleles associated with the presence of anti-topo I antibody in various ethnicities and their amino acid sequence at positions 67–71 in the third hypervariable region (HVR). **B** Proliferative responses of a representative topo I-reactive CD4^+^ T cell clone derived from an anti-topo I-positive patient with SSc in the presence of antigenic peptide-pulsed L cell transfectants expressing a series of human HLA-DR molecules harboring the *DRB1* or *DRB5* alleles associated with the presence of anti-topo I antibody. The peptide-induced T cell proliferation was measured by ^3^H-thymidine incorporation

A number of studies have found that HLA class II genes are associated with SSc-related autoantibodies rather than SSc itself, while the associated HLA class II alleles are different among ethnic groups [96]. The association of HLA class II alleles with SSc-related autoantibodies were analyzed most intensively for the anti-topo I antibody. It was reported that anti-topo I was associated with DQB1*03, which was thought to play the primary role in Caucasians [97]. On the other hand, we found that the DRB1*15;02, DRB5*01;02, DQB1*06;01 haplotype was associated with anti-topo I-positive SSc in Japanese population [98]. Since the *DRB1/B5* alleles associated with anti-topo I in various ethnic groups, DRB1*11;04 in Caucasians, DRB1*08;04/*11;01 in American Africans, DRB1*11;04/*08;02 in Hispanics, and DRB5*01;02 in Japanese, have the common amino acid sequence FLEDR at amino acid positions at 67–71 in the hypervariable β1 domain of the *DRB* gene, we have proposed that the *DRB* is the primary gene associated with anti-topo I antibody in SSc patients (Fig. 1A). Full-blooded Choctaw Native Americans living in southeastern Oklahoma have the highest prevalence of SSc [99]. Anti-topo I antibody is the predominant autoantibody in this patent population and is associated with the unique Amerindian HLA haplotype containing DRB1*1602, which has the ILEDR sequence at amino acid positions at 67–71 [93]. Since antigen-presenting cells present an antigenic peptide to CD4^+^ T cells in the context of HLA class II molecules, it is likely that the amino acid sequence FLEDR at positions 67–71 of the *DRB* gene located at the bottom of the antigen-binding groove controls antigen-specific CD4^+^ T cell responses. To test this hypothesis, we examined in vitro T cell proliferative response to recombinant topo I fragments and found that the HLA-DR-restricted T cell response was found in SSc patients with anti-topo I as well as in heathy controls who had the *DRB* alleles with the FLEDR sequence [93]. We further established topo I-reactive CD4^+^ T cell clones and examined their HLA class II restriction using a series of mouse L cell transfectants pulsed with the antigenic topo I peptide [100]. The antigen-induced response of the T cell clones was observed upon a co-culture with L cell transfectants expressing the DRA molecule in combination with DRB molecules harboring DRB1*11;01, *08;02, or DRB5*01;02 (Fig. 1B), indicating that the FLEDR sequence was critical to present the antigenic topo I peptide and resultant CD4^+^ T cell activation, potentially leading to anti-topo I autoantibody production. A recent 3D structure models for prediction of HLA α/β heterodimers using the associated amino acid residues in the peptide-binding groove successfully identified immunodominant peptides of topo I [101]. On the other hand, previous studies have proposed that the *DPB1* locus is the primary susceptibility gene for anti-topo I-positive SSc within the HLA region and DPB1*13:01 is strongly associated with SSc and anti-topo I antibody in various ethnic groups [97, 101]. The DPB1*13:01 is linked with a variety of *DRB1* alleles with low linkage disequilibrium value, i.e., DRB1*01;01, 15;01, 04;06, *11;01*, and *12;01* in Japanese, suggesting that association of DPB1*13:01 with anti-topo I antibody-positive SSc is independent of the *DRB1/B5* allele association with production of anti-topo I antibody. Interestingly, the recent high-density microsatellite analysis of the HLA region identified *RXRB* as the responsible gene on the risk haplotype harboring HLA-DPB1*13:01 in SSc patients with anti-topo I antibody in Japanese population [91].

Many lines of evidence have shown that individual SSc-related autoantibodies have associations with different HLA class II alleles and haplotypes, including ACA with the DRB1*01;01, DQB1*05;01 haplotype, anti-RNA polymerase III with DRB1*04;01/*04;04, anti-PM-Scl with the DRB1*03;01, DQB1*02;01 haplotype, and anti-U1RNP with the DRB1*04;01, DQB1*03;02 haplotype [94, 98, 102]. These strong links could explain difference in prevalence of individual SSc-related autoantibodies among ethnic groups: anti-RNA polymerase III was more prevalent in cohorts from the UK, Northeast USA, and Australia, compared with other European countries and Japan [103], while anti-PM-Scl is almost exclusively found in Caucasian patients with SSc [104]. Conditional analysis in the autoantibody subsets of SSc revealed several associated amino acid residues, mostly in the peptide-binding groove of the HLA class II molecules [97]. It is interesting to note that bioinformatically predicted immunodominant peptides of topo I, fibrillarin, and centromere protein A are homologous to viral protein sequences, suggesting a possible link between HLA alleles, autoantibodies, and environmental triggers in the pathogenesis of SSc.

## Gene expression profiling of the affected organ systems

The technologies to analyze gene expression profiling have enabled us to evaluate expression levels of comprehensive genes in peripheral blood, skin, and other affected tissues in patients with SSc. The gene expression analysis using microarray on skin biopsies from patients with SSc or morphea and healthy controls found four unique expression patterns in SSc patients: “fibro-proliferative”, “inflammatory”, “limited”, and “normal-like” [105]. The fibro-proliferative pattern comprised of patients with dcSSc, with the gene set associated with the biological processes of cell cycle. The inflammatory pattern was characterized by increased expression of a series of immune response genes. The limited pattern was predominantly found in lcSSc patients with a high expression of a distinct signature found heterogeneously across the samples. Lastly, the normal-like pattern had increased expression of genes associated with fatty acid metabolism and lacked any expression associated with inflammation or proliferation. The gene expression profiling in the affected skin has called attention because this information is useful as biomarkers for predicting progression of the disease and treatment responses. For example, Hinchcliff et al. found that patients who responded to mycophenolate mofetil predominantly had inflammatory pattern, whereas all patients with the fibro-proliferative pattern were non-responders [106]. In a phase IIb, placebo-controlled, randomized clinical trial of abatacept in patients with early dcSSc, there was no statistically significant difference in changes of modified Rodnan total skin thickness score (mRSS) at 52 weeks in patients treated with abatacept compared with those treated with placebo [107]. Interestingly, in a subgroup analysis, abatacept significantly improved mRSS compared with placebo in patients with inflammatory pattern of gene expression in the skin, but not in those with other gene expression patterns.

## Conclusions and future perspectives

Recent advances of technology of the genetic study such as GWAS have successfully identified a number of associations between genetic polymorphisms and SSc. A series of studies have suggested that dysregulated innate and adaptive immunity linked to genetic predisposition is involved in the pathogenic process of SSc but is insufficient to fully elicit microvasculopathy and excessive fibrosis, which are characteristics to SSc. We now know that estimated heritability of SSc is lower than other autoimmune diseases, such as RA and SLE, and contribution of environmental factors and epigenetic influences is more important in the development of SSc [108]. Acquired alteration in processes involved in DNA methylation and histone modification, and dysregulated miRNA network plays a critical role in the development of SSc, although the pathways linked between genetic factors and environmental triggers are still not fully understood. Therefore, the multi-omics analyses, including transcriptome, proteome, and metabolome will open up new avenues for improving understanding of the complex molecular architecture of SSc, predicting outcomes and treatment responses, and discovering new drug targets.

## Abbreviations

SSc: Systemic sclerosis
topo I: Topoisomerase I
HLA: Human leukocyte antigen
RA: Rheumatoid arthritis
SLE: Systemic lupus erythematosus
ANA: Anti-nuclear antibody
ILD: Interstitial lung disease
SNP: Single-nucleotide polymorphism
VNTR: Variable number of tandem repeat
CNV: Copy-number variation
CGA: Candidate gene approach
GWAS: Genome-wide association study
eQTL: Expression quantitative trait loci
IFN: Interferon
IRF: Interferon regulatory factor
IL: Interleukin
TNF: Tumor-necrosis factor
dcSSc: Diffuse cutaneous systemic sclerosis
ACA: Anticentromere antibody
TNFAIP3: Tumor-necrosis factor-α-induced protein 3
α-SMA: α-Smooth muscle actin
TGF-β: Transforming growth factor-β
TNIP1: Tumor-necrosis factor-α-induced protein 3-interacting protein 1
TNFSF4: Tumor-necrosis factor ligand superfamily member 4
TCR: T cell receptor
PTPN22: Protein tyrosine phosphatase, non-receptor type 22
LYP: Lymphoid tyrosine phosphatase
CSK: c-Src kinase
STAT4: Signal transducer activator transcriptional factor 4
Th1: T helper 1
lcSSc: Limited cutaneous systemic sclerosis
IL-12RB: Interleukin-12 receptor B
IL-2RA: Interleukin 2 receptor subunit alpha
BANK1: B cell-specific scaffold protein with ankyrin 1
BLK: B lymphocyte kinase
PRDM1: B lymphocyte-induced maturation protein 1
DNASE1L3: Deoxyribonuclease 1-like 3
GRB10: Growth factor receptor-bound protein 10
ATG5: Autophagy-related 5
RAB2A: Ras-related protein Rab-2A
GSDMA: Gasdermin A
NOTCH4: Neurogenic locus notch homolog protein 4
PPARG: Peroxisome proliferator-activated receptor γ
CAV1: Caveolin 1
RXRB: Rare variant of retinoid X receptor-beta
mRSS: Modified Rodnan total skin thickness score
HVR: Hypervariable region
